# Supplementary material for: Dynamic functional connectivity changes in dementia with Lewy bodies and Alzheimer's disease
Source: Neuroimage Clin. 2019 Apr 3;22:101812. doi: 10.1016/j.nicl.2019.101812 (PMC6462776; doi:10.1016/j.nicl.2019.101812)
Supplement: Supplementary file 1 — Supplementary material [file mmc1.docx]

Supplementary Material: Dynamic functional connectivity changes in dementia with Lewy bodies and Alzheimer’s disease

Julia Schumacher, Luis R. Peraza, Michael Firbank, Alan J. Thomas, Marcus Kaiser, Peter Gallagher, John T. O’Brien, Andrew M. Blamire, John-Paul Taylor

Contents

1. Independent healthy control group
2. Locations of RSN spatial maps
3. Standard deviation for different window sizes
4. Effect sizes for group comparison of dynamic connectivity
5. K-means evaluation

5.1. Choice of number of clusters

5.2. Statistics for group comparison of k-means characteristics

5.3. Results for different values of k

5.4. Results for k=3 and different window sizes
5.5. Bootstrap and split-half resampling

1. Correlations with clinical scores
2. Effect of dopaminergic medication in the DLB group
3. Relation between motion and dynamic connectivity measures
4. Group differences in grey matter volume
5. Effect of grey matter atrophy on dynamic connectivity measures

1. Independent healthy control group

Table S1: Demographics of independent healthy control group, compared to control group from main analysis

|  | HC main analysis  (N=31) | HC for RSN template estimation  (N=42) | Between-group comparison |
| --- | --- | --- | --- |
| Male: female | 22:9 | 25:17 | χ^2^=1.02, p=0.31 |
| Age | 76.4 (7.2) | 69.0 (8.7) | t_70_=3.85, p<0.001 |
| MMSE | 28.9 (1.1) | 29.2 (1.4) | t_70_=1.08, p=0.29 |
|  |  |  |  |

HC, healthy controls; MMSE, Mini Mental State Examination

To estimate independent healthy resting state networks (RSNs), 44 healthy older adult controls (HC) from two previous studies were selected. They were significantly younger than the HCs from the main analysis, but matched in terms of overall cognition (Table S1).
All participants were scanned on the same scanner as the participants from the main analysis. Eighteen of the additional HC participants were scanned with a slightly different scanner protocol with a change in the TR to 2072 ms and a change in the voxel size of the resting state scans to 3 x 3 x 4 mm^3^.
The resting state data were preprocessed in the same way as described in the main manuscript. Two subjects were excluded because they exceeded the motion exclusion criteria resulting in 42 independent HC participants that were included in the generation of the RSN templates.

2. Locations of RSN spatial maps

Table S2: List of all resting state networks included in the analysis. Anatomical labels refer to bilateral areas if not stated otherwise. Locations of RSNs are estimated from the Harvard-Oxford Cortical and Subcortical Structural Atlases and the Cerebellar Atlas in FSL.

| RSN name |  | Brain regions |
| --- | --- | --- |
| Lateral sensorimotor network | LSMN | Pre- and postcentral gyrus |
| Medial sensorimotor network | MSMN | Pre- and postcentral gyrus, supplementary motor area |
| Supplementary motor area network | SMAN | Supplementary motor area, precentral gyrus |
| Left motor network | LMN | Left post- and precentral gyrus |
| Right motor network | RMN | Right post- and precentral gyrus |
| Basal ganglia network | BGN | Putamen, caudate |
| Thalamic network | THN | Thalamus |
| Cerebellar network 1 | CBN1 | Cerebellum crus I, crus II |
| Cerebellar network 2 | CBN2 | Cerebellum V, VI |
| Medial visual network | MVN | Intracalcarine cortex, supracalcarine cortex, lingual gyrus |
| Lateral visual network | LVN | Superior lateral occipital cortex, precuneus |
| Occipital pole network | OPN | Occipital pole |
| Lingual gyrus network | LGN | Lingual gyrus, intracalcarine cortex |
| Superior visual network | SVN | Superior lateral occipital cortex, occipital pole |
| Temporal network | TN | Planum temporale, Heschl’s gyrus |
| Temporal pole network | TPN | Temporal pole |
| Insular network 1 | ISN1 | Insular cortex, frontal operculum cortex |
| Insular network 2 | ISN2 | Insular cortex, planum polare |
| Anterior cingulate network | ACN | Anterior cingulate cortex |
| Default mode network 1 | DMN1 | Precuneus, posterior cingulate cortex |
| Default mode network 2 | DMN2 | Precuneus |
| Default mode network 3 | DMN3 | Precuneus, superior lateral occipital cortex |
| Supramarginal gyrus network | SPGN | Supramarginal gyrus |
| Right fronto-parietal network | RFPN | Right superior lateral occipital cortex, right angular gyrus, right middle frontal gyrus, left superior lateral occipital cortex |
| Left fronto-parietal network | LFPN | Left superior lateral occipital cortex, right angular gyrus, left middle frontal gyrus, right superior lateral occipital cortex |
| Dorsal attention network | DAN | Superior parietal lobule, supramarginal gyrus, superior lateral occipital cortex |
| Ventral attention network | VAN | Middle frontal gyrus, inferior frontal gyrus |

3. Standard deviation for different window sizes
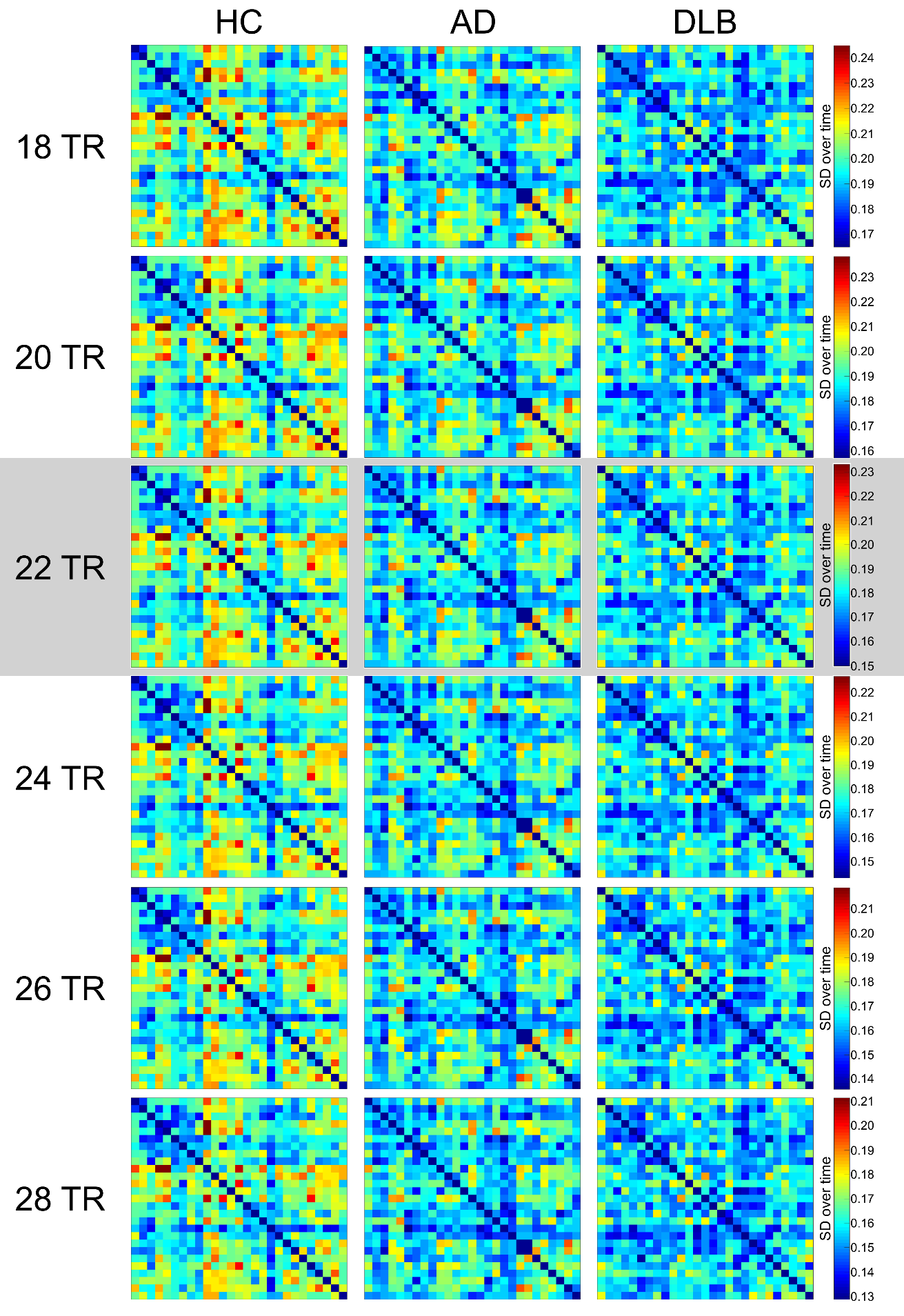


Figure S1: Mean standard deviation matrices for healthy controls (HC), Alzheimer’s disease (AD), and dementia with Lewy bodies (DLB) for different window sizes ranging from 18 TR to 28 TR. The grey box highlights the solution presented in the main text.

4. Effect sizes for group comparison of dynamic connectivity

Table S3: Effect sizes using non-parametric r^2^ for comparing the mean SD overall and for each network separately between groups. r^2^ is multiplied by 100 and corresponds to the percentage of variance of the dependent variable that is explained by the independent variable.
r^2^=Z^2^/N with N=total group size of the respective comparison and Z score from post-hoc Dunn’s test.

| RSN name | HC-AD r^2^ | HC-DLB r^2^ | AD-DLB r^2^ |
| --- | --- | --- | --- |
| Overall mean SD | 3.05 | 7.12 | 0.85 |
| LSMN | 0.80 | 0.20 | 0.20 |
| MSMN | 3.75 | 1.71 | 0.40 |
| SMAN | 1.74 | 0.91 | 0.13 |
| LMN | 0.66 | 4.52 | 1.72 |
| RMN | 0.58 | 4.49 | 1.84 |
| BGN | 0.01 | 2.56 | 2.22 |
| THN | 0.92 | 0.07 | 0.48 |
| CBN1 | 0.88 | 1.24 | 0.03 |
| CBN2 | 1.38 | 2.94 | 0.29 |
| MVN | 2.52 | 9.97 | 2.46 |
| LVN | 6.91 | 7.99 | 0.04 |
| OPN | 0.19 | 0.09 | 0.02 |
| LGN | 2.23 | 6.58 | 1.15 |
| SVN | 1.86 | 7.99 | 2.13 |
| TN | 2.28 | 4.41 | 0.35 |
| TPN | 9.74 | 7.92 | 0.09 |
| ISN1 | 0.94 | 0.66 | 0.03 |
| ISN2 | 0.41 | 0.70 | 0.04 |
| ACN | 3.03 | 6.77 | 0.74 |
| DMN1 | 1.01 | 3.58 | 0.79 |
| DMN2 | 0.14 | 3.94 | 2.61 |
| DMN3 | 1.35 | 1.87 | 0.04 |
| SPGN | 0.30 | 5.47 | 3.21 |
| RFPN | 2.62 | 1.37 | 0.20 |
| LFPN | 2.24 | 8.41 | 1.96 |
| DAN | 1.01 | 6.00 | 2.08 |
| VAN | 4.42 | 2.44 | 0.29 |


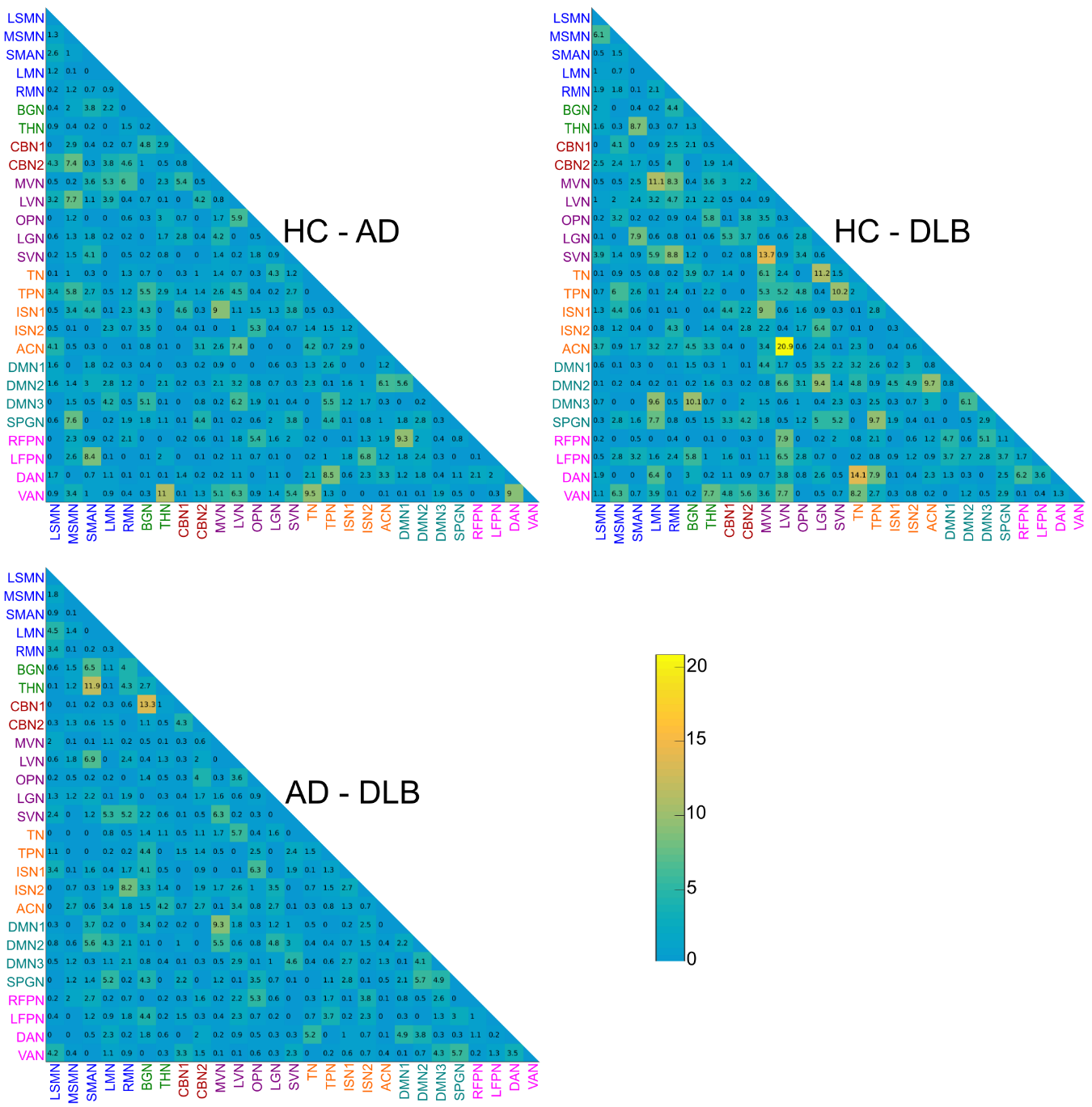


Figure S2: Effect sizes using non-parametric r^2^ for comparing each RSN-to-RSN connection between the groups separately. r^2^ is multiplied by 100 and corresponds to the percentage of variance of the dependent variable that is explained by the independent variable.
r^2^=Z^2^/N with N=total group size of the respective comparison and Z score from post-hoc Dunn’s test.

5. K-means evaluation

5.1. Choice of number of clusters


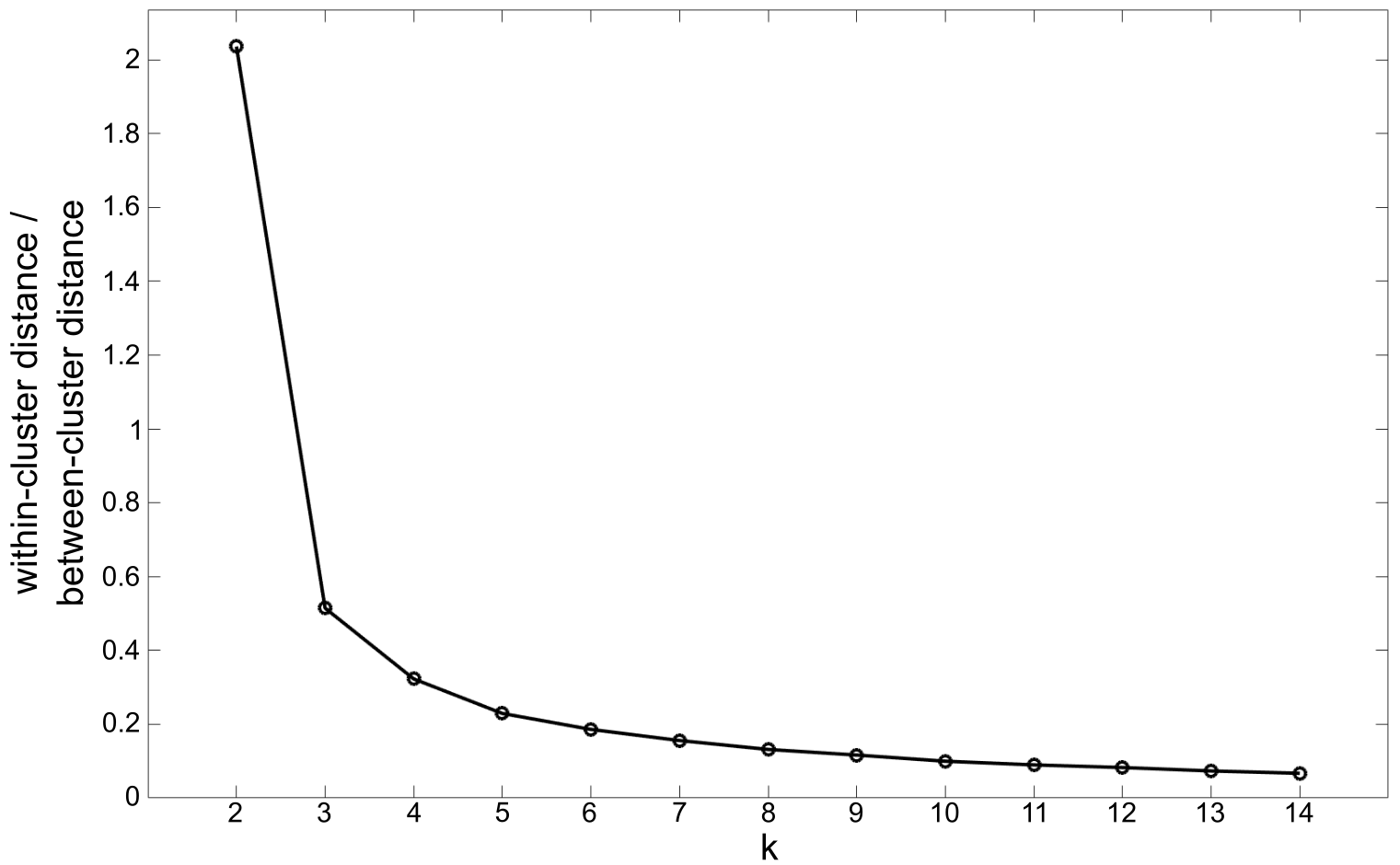


Figure S3: Elbow plot of the cluster validity index, i.e. ratio of within-cluster distance to between-cluster distance, for k=2 to 14.

5.2. Statistics for group comparison of k-means characteristics and network measures

Table S4: Results from overall non-parametric multivariate ANOVA (MANOVA) and follow-up Kruskal-Wallis ANOVAs per state for group comparison of k-means characteristics and static and dynamic efficiency measures between HC, AD, and DLB. Correlations between time and the occurrence of the three states were calculated using Spearman’s rank correlations.

|  | | H/F | p, uncorrected | p, FDR-corrected |
| --- | --- | --- | --- | --- |
| Frequency | MANOVA | 2.69 | 0.034 | - |
|  | State 1 | 8.61 | 0.013 | 0.040 |
|  | State 2 | 6.95 | 0.031 | 0.046 |
|  | State 3 | 0.98 | 0.61 | 0.61 |
| Mean dwell time | MANOVA | 2.85 | 0.023 | - |
|  | State 1 | 8.49 | 0.014 | 0.043 |
|  | State 2 | 6.05 | 0.048 | 0.072 |
|  | State 3 | 2.71 | 0.26 | 0.26 |
| Number of transitions | | 3.16 | 0.21 | - |
| Intertransition interval | | 3.21 | 0.20 | - |
| SD of local efficiency | | 0.89 | 0.64 | - |
| SD of global efficiency | | 6.08 | 0.047 | - |
| static local efficiency | | 2.30 | 0.32 | - |
| static global efficiency | | 2.01 | 0.37 | - |
| Correlation with time | | ρ | p, uncorrected | p, FDR-corrected |
| Occurrence of state 1 | | -0.03 | 0.78 | 0.78 |
| Occurrence of state 2 | | 0.05 | 0.62 | 0.78 |
| Occurrence of state 3 | | -0.19 | 0.045 | 0.13 |

Table S5: Effect sizes for group comparison of k-means characteristics and static and dynamic efficiency measures using r^2^. r^2^ is multiplied by 100 and corresponds to the percentage of variance of the dependent variable that is explained by the independent variable.
r^2^=Z^2^/N with N=total group size of the respective comparison and Z score from post-hoc Dunn’s test.

|  | | mean (SD) | | | HC-AD | HC-DLB | AD-DLB |
| --- | --- | --- | --- | --- | --- | --- | --- |
|  |  | HC | AD | DLB | r^2^ | r^2^ | r^2^ |
| Frequency | State 1 | 0.31 (0.39) | 0.10 (0.20) | 0.05 (0.12) | 9.03 | 11.77 | 0.18 |
|  | State 2 | 0.38 (0.38) | 0.52 (0.35) | 0.63 (0.31) | 4.17 | 11.03 | 1.63 |
|  | State 3 | 0.31 (0.35) | 0.37 (0.35) | 0.32 (0.31) | 1.56 | 0.73 | 0.16 |
| Mean dwell time | State 1 | 20.2 (27.6) | 6.11 (11.5) | 4.11 (8.21) | 8.99 | 11.53 | 0.16 |
|  | State 2 | 27.5 (34.3) | 38.6 (36.1) | 39.7 (31.3) | 5.01 | 9.07 | 0.60 |
|  | State 3 | 25.4 (35.4) | 27.7 (30.6) | 17.0 (20.0) | 3.70 | 0.02 | 3.14 |
| Number of transitions | | 2.5 (2.0) | 2.4 (1.8) | 3.2 (1.8) | 0.05 | 3.40 | 4.31 |
| Intertransition interval | | 44.3 (33.5) | 47.7 (37.7) | 34.6 (29.6) | 0.06 | 3.45 | 4.37 |
| SD of local efficiency | | 0.030 (0.010) | 0.027 (0.009) | 0.024 (0.008) | 1.24 | 0.93 | 0.02 |
| SD of global efficiency | | 0.032 (0.007) | 0.034 (0.009) | 0.034 (0.008) | 1.99 | 9.77 | 2.94 |
| static local efficiency | | 0.51 (0.4) | 0.49 (0.04) | 0.49 (0.03) | 3.52 | 1.90 | 0.25 |
| static global efficiency | | 0.46 (0.03) | 0.46 (0.03) | 0.47 (0.02) | 0.90 | 3.24 | 0.73 |

5.3. Results for different values of k

**
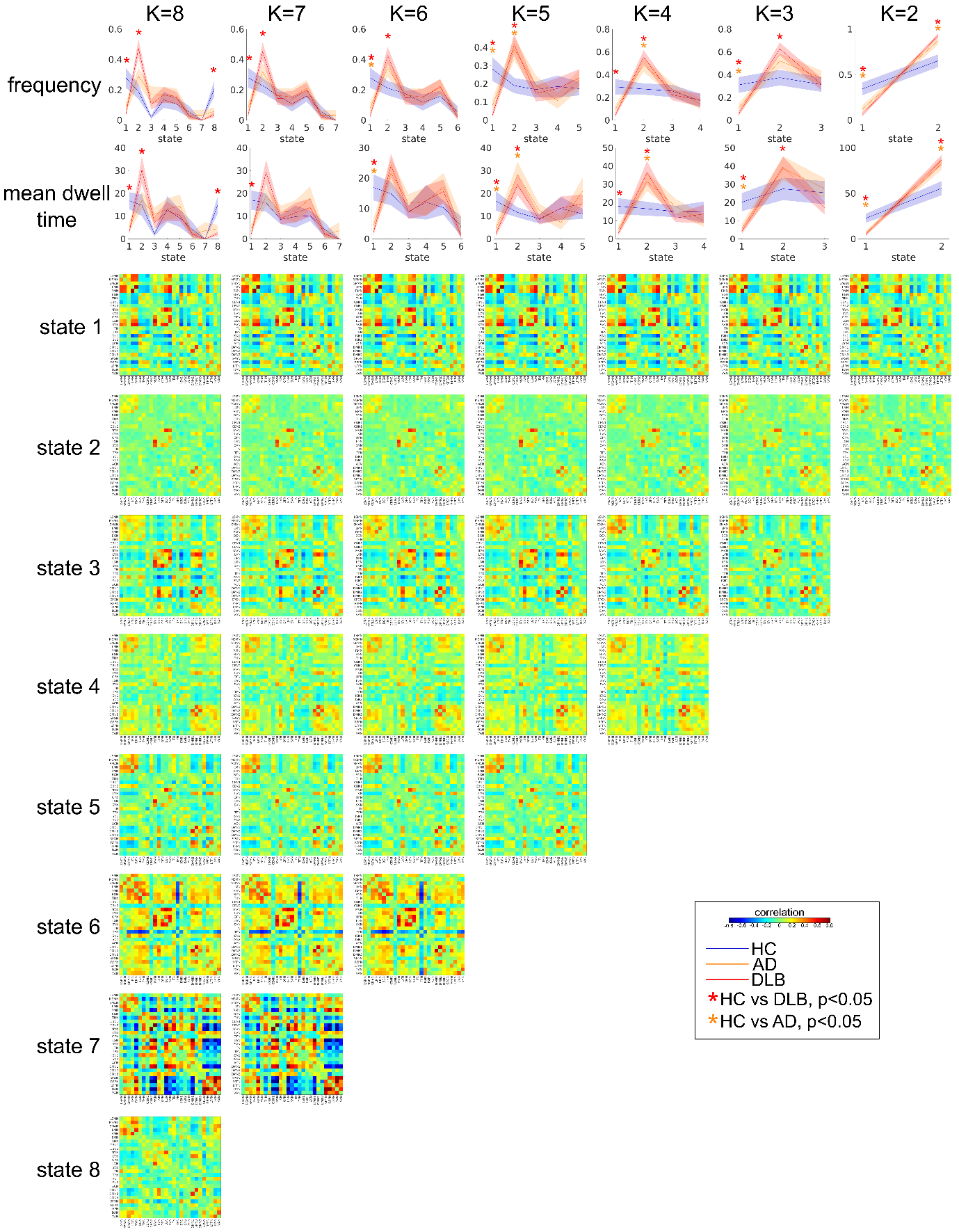
**

Figure S4: Results from k-means analysis (frequency and dwell time for the different states) for different values of k.

5.4. Results for k=3 and different window sizes

**
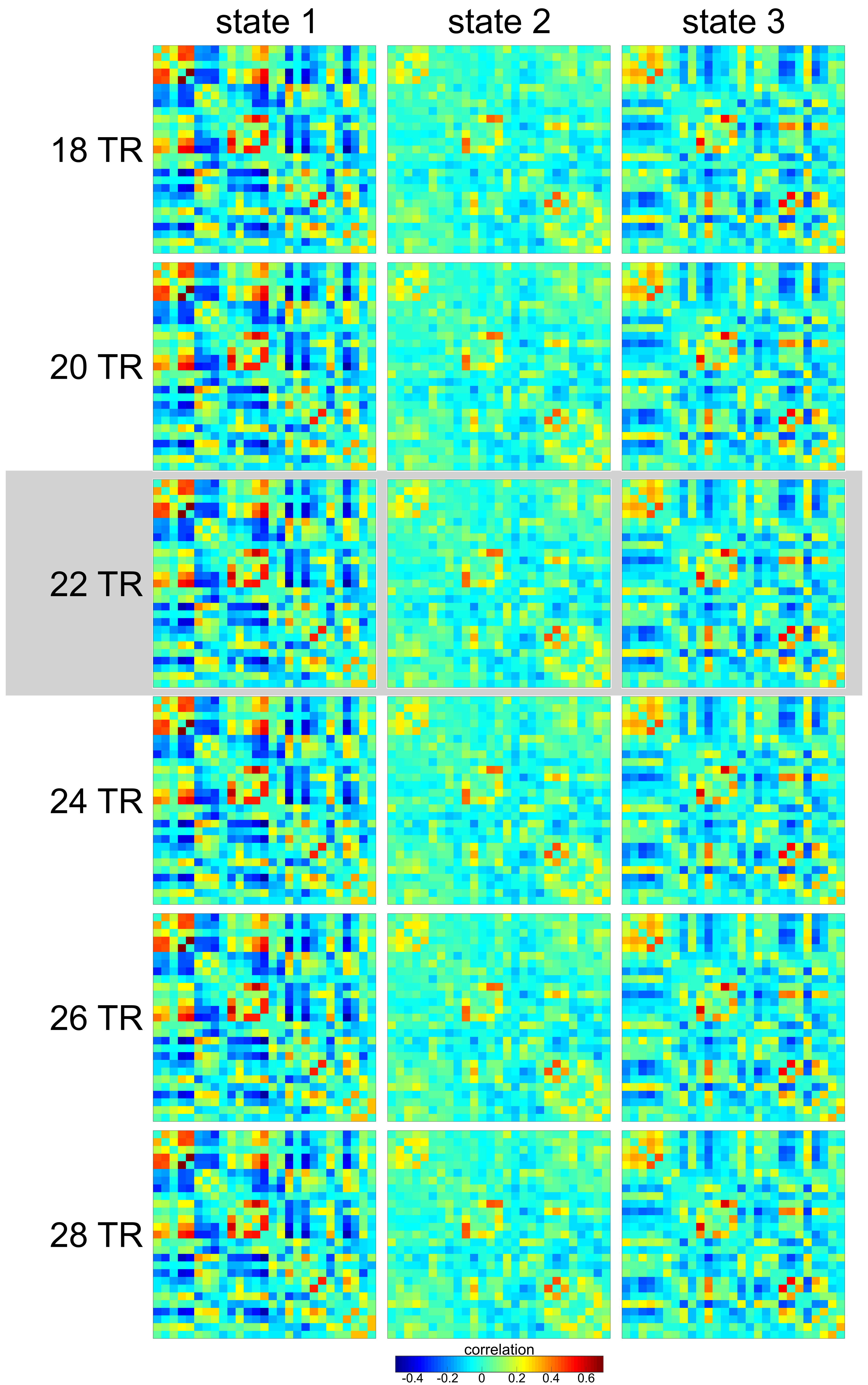
**

Figure S5: States from k-means analysis with k=3 for different window sizes ranging from 18 TR to 28 TR. The grey box highlights the solution presented in the main text.

5.5. Bootstrap and split-half resampling


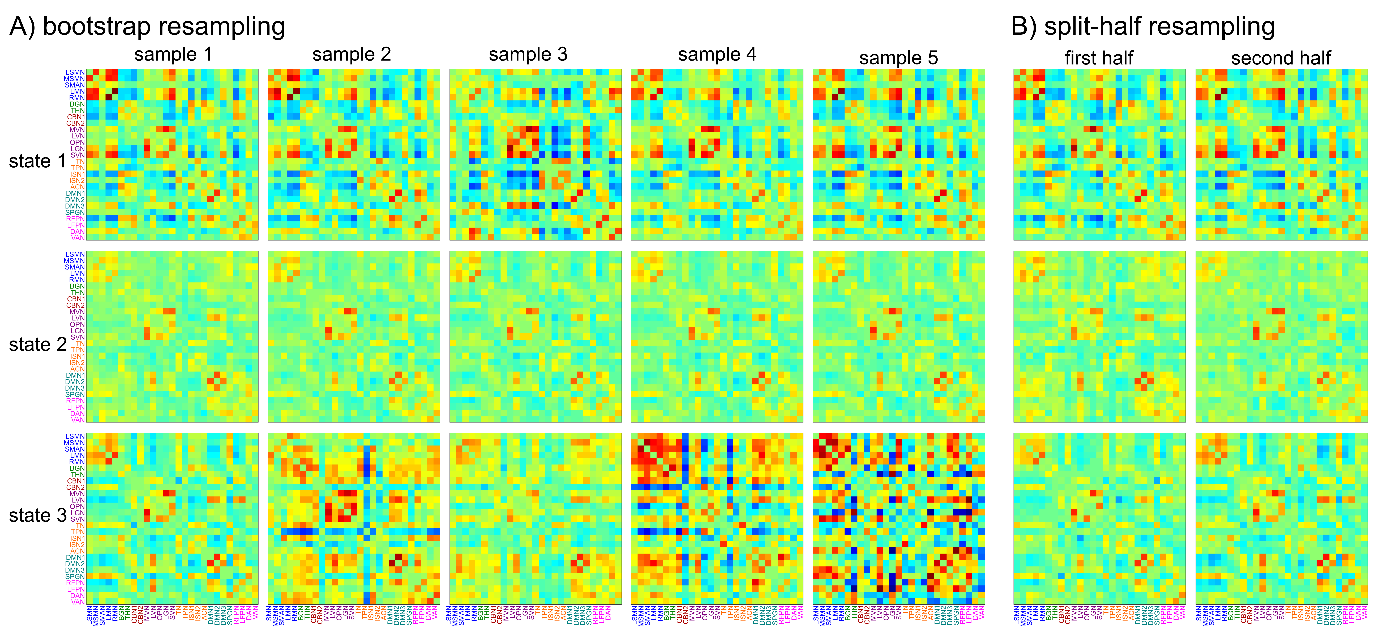


Figure S6: K-means analysis with k=3 on A) bootstrapped resamples of the data, B) splithalf samples. Bootstrapping was performed by randomly selecting 31 HC, 29 AD, and 31 DLB participants with replacement and was repeated five times. Split-half resampling was performed by splitting the whole group of participants in half with the constraint that each half contained approximately the same number of participants from each of the three groups.

6. Correlations with clinical scores in DLB

Table S6: Spearman’s rank correlation between clinical scores and dynamic connectivity measures that show differences between controls and dementia patients. Correlations are calculates for overall cognition (MMSE, CAMCOG) in the DLB and AD groups and for DLB-specific symptoms (UPDRS, CAF, NPI hallucinations) in the DLB group.

|  | ρ | p, uncorrected | p, FDR-corrected |
| --- | --- | --- | --- |
| DLB |  |  |  |
| UPDRS – frequency state 1 | -0.135 | 0.468 | 0.88 |
| UPDRS – frequency state 2 | 0.389 | 0.031 | 0.67 |
| UPDRS – dwell time state 1 | -0.099 | 0.596 | 0.88 |
| UPDRS – dwell time state 2 | 0.160 | 0.391 | 0.88 |
| UPDRS – SD global efficiency | -0.019 | 0.919 | 0.95 |
| CAF total^a^ – frequency state 1 | 0.145 | 0.444 | 0.88 |
| CAF total^a^ – frequency state 2 | 0.065 | 0.732 | 0.95 |
| CAF total^a^ – dwell time state 1 | 0.118 | 0.535 | 0.88 |
| CAF total^a^ – dwell time state 2 | 0.127 | 0.503 | 0.88 |
| CAF total^a^ – SD global efficiency | 0.011 | 0.953 | 0.95 |
| NPI hall^b^ – frequency state 1 | 0.014 | 0.942 | 0.95 |
| NPI hall^b^ – frequency state 2 | -0.279 | 0.143 | 0.88 |
| NPI hall^b^ – dwell time state 1 | -0.013 | 0.945 | 0.95 |
| NPI hall^b^ – dwell time state 2 | -0.319 | 0.092 | 0.88 |
| NPI hall^b^ – SD global efficiency | 0.035 | 0.858 | 0.95 |
| MMSE – frequency state 1 | -0.142 | 0.445 | 0.88 |
| MMSE – frequency state 2 | -0.079 | 0.673 | 0.92 |
| MMSE – dwell time state 1 | -0.158 | 0.397 | 0.88 |
| MMSE – dwell time state 2 | -0.098 | 0.602 | 0.88 |
| MMSE – SD of global efficiency | 0.248 | 0.178 | 0.88 |
|  |  |  |  |
| AD |  |  |  |
| MMSE – frequency state 1 | 0.113 | 0.559 | 0.88 |
| MMSE – dwell time state 1 | 0.116 | 0.549 | 0.88 |

^a^ N=30, ^b^ N=29

7. Effect of dopaminergic medication in the DLB group

Table S7: To assess the effect of the use of dopaminergic medication in a subset of the DLB patients on dynamic connectivity measures, a group comparison was performed between patients who were on dopaminergic medication (N=18) and those patients not taking dopaminergic medication (N=13) using independent samples Mann-Whitney U-tests.

|  | mean (SD) | | U | p-value |
| --- | --- | --- | --- | --- |
|  | DLB on PD meds (N=18) | DLB not on PD meds (N=13) |  |  |
| overall SD | 0.18 (0.01) | 0.18 (0.01) | 111.00 | 0.83 |
| frequency state 1 | 0.04 (0.08) | 0.07 (0.16) | 111.00 | 0.83 |
| frequency state 2 | 0.65 (0.36) | 0.59 (0.23) | 95.50 | 0.40 |
| frequency state 3 | 0.31 (0.37) | 0.33 (0.22) | 90.00 | 0.30 |
| mean dwell time state 1 | 4.22 (8.30) | 3.95 (8.42) | 115.00 | 0.95 |
| mean dwell time state 2 | 43.38 (36.05) | 34.50 (23.68) | 112.00 | 0.86 |
| mean dwell time state 3 | 17.78 (25.78) | 16.03 (7.72) | 82.50 | 0.17 |
| number of transitions | 2.67 (1.88) | 3.85 (1.52) | 75.00 | 0.10 |
| intertransition interval | 41.65 (36.40) | 24.77 (11.50) | 88.50 | 0.26 |
| SD of local efficiency | 0.04 (0.006) | 0.03 (0.01) | 86.00 | 0.23 |
| SD of global efficiency | 0.02 (0.009) | 0.02 (0.005) | 110.00 | 0.80 |

8. Relation between motion and dynamic connectivity measures

Table S8: To investigate whether the dynamic connectivity results were influenced by motion, we calculated correlations between the dynamic connectivity measures and mean framewise displacement across all participants using Spearman’s correlations.

|  | ρ | p-value |
| --- | --- | --- |
| frequency state 1 | -0.08 | 0.43 |
| frequency state 2 | 0.14 | 0.18 |
| mean dwell time state 1 | -0.06 | 0.56 |
| mean dwell time state 2 | 0.12 | 0.27 |
| SD of local efficiency | 0.07 | 0.49 |
| SD of global efficiency | -0.09 | 0.42 |

9. Group differences in grey matter volume


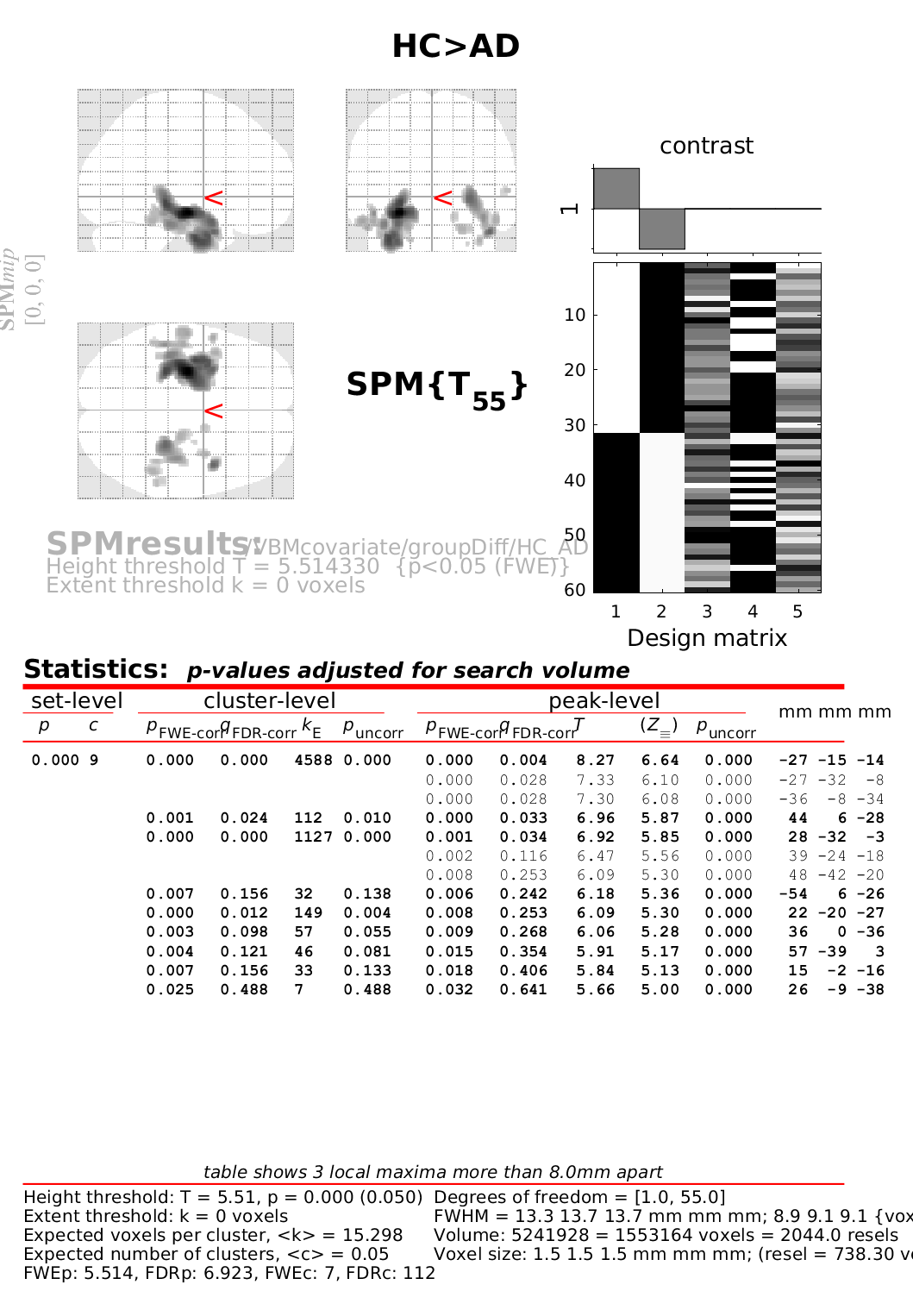


Figure S7: Results from VBM analysis comparing AD and controls.


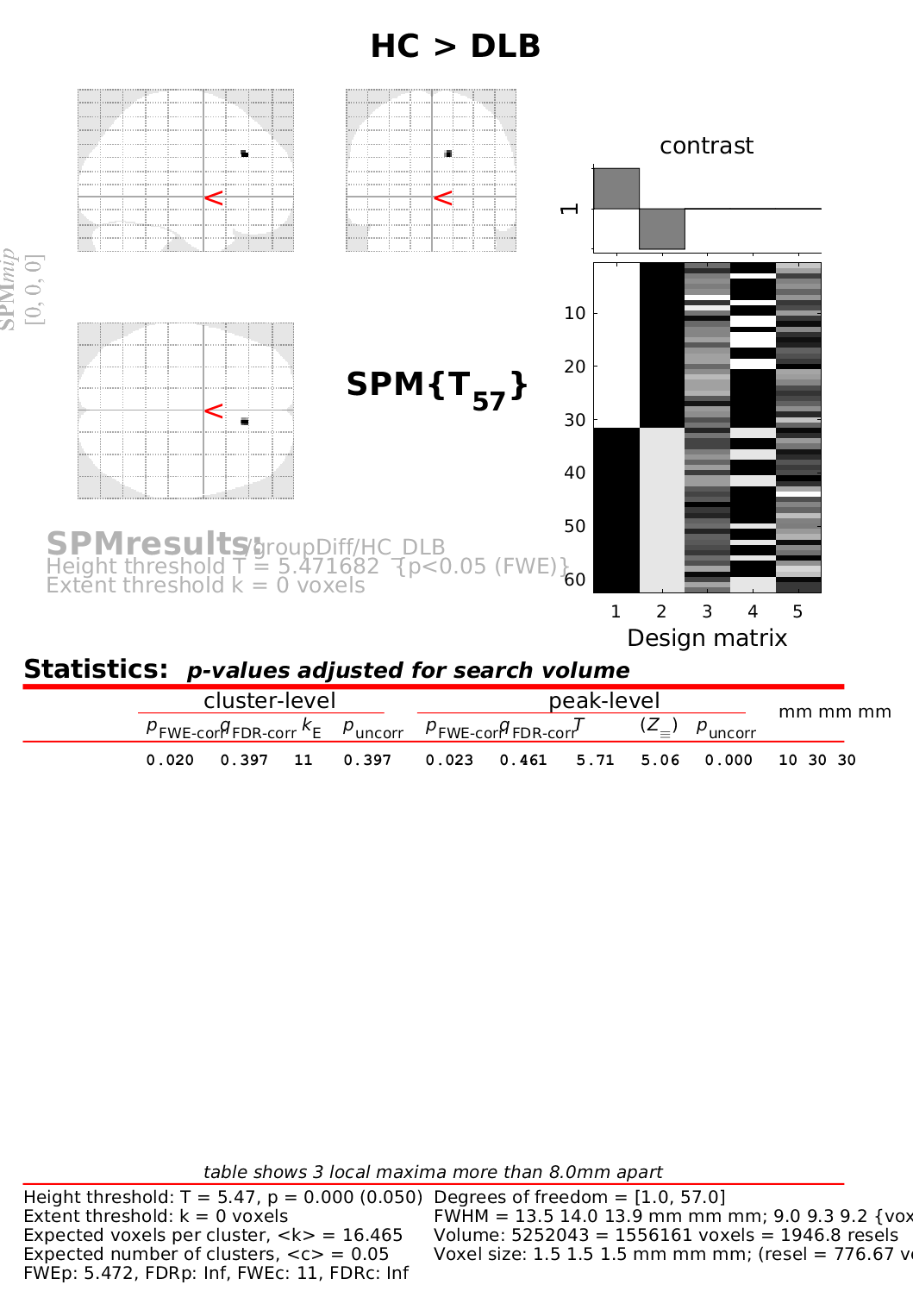


Figure S8: Results from VBM analysis comparing DLB and controls.

10. Effect of grey matter atrophy on dynamic connectivity measures


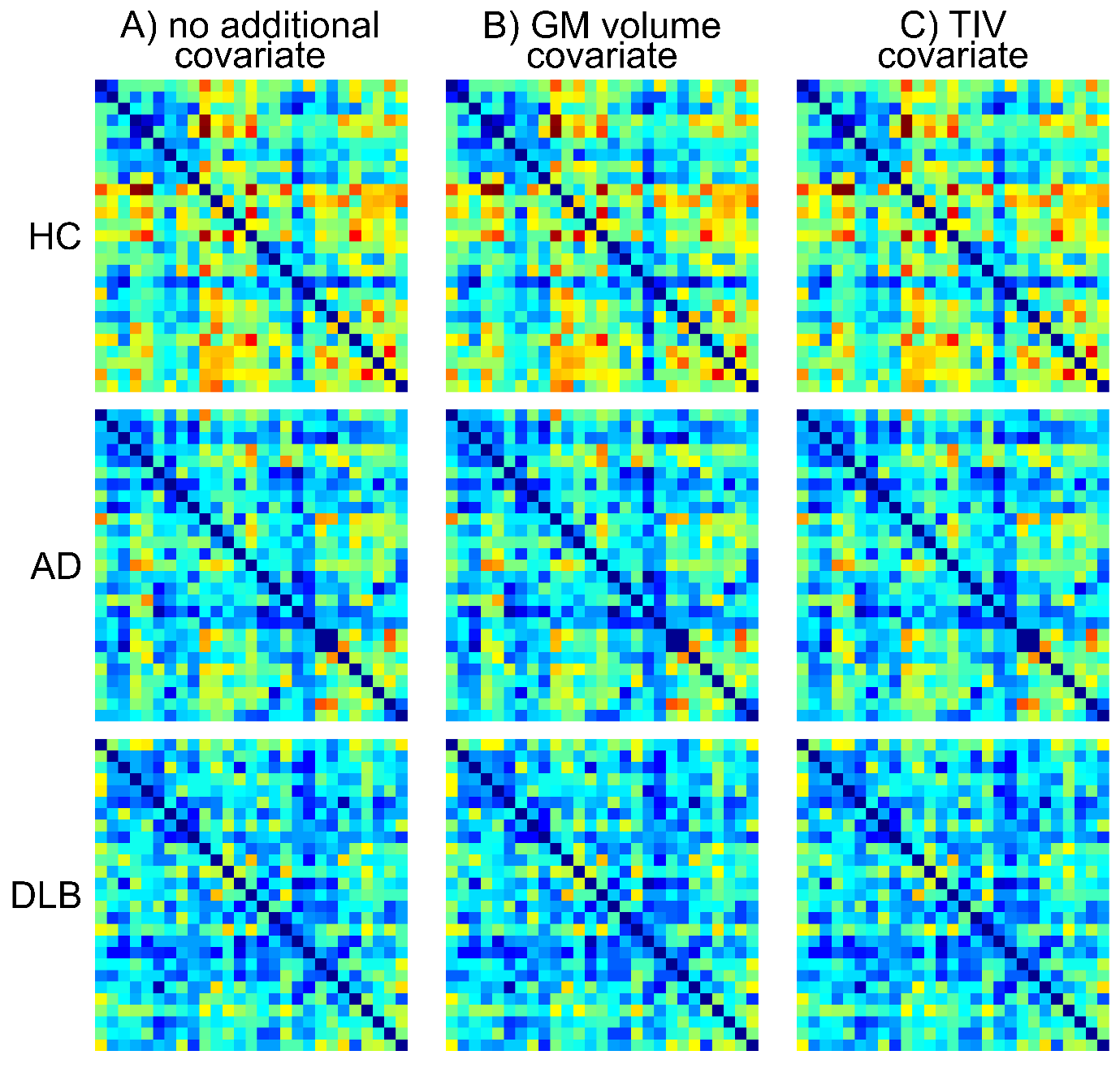


Figure S9: Effect of adding different covariates when residualizing the windowed correlation matrices (see Section 2.5 of the main text). A) SD matrices for HC, AD, and DLB using only the covariates that were used in the main text (age, gender, study) for comparison (see Figure 3 of the main text). B) SD matrices for HC, AD, and DLB after adding a covariate for total grey matter (GM) volume estimated by SPM. C) SD matrices for HC, AD, and DLB after adding a covariate for total intracranial volume (TIV=GM volume+ white matter volume + CSF) estimated by SPM.


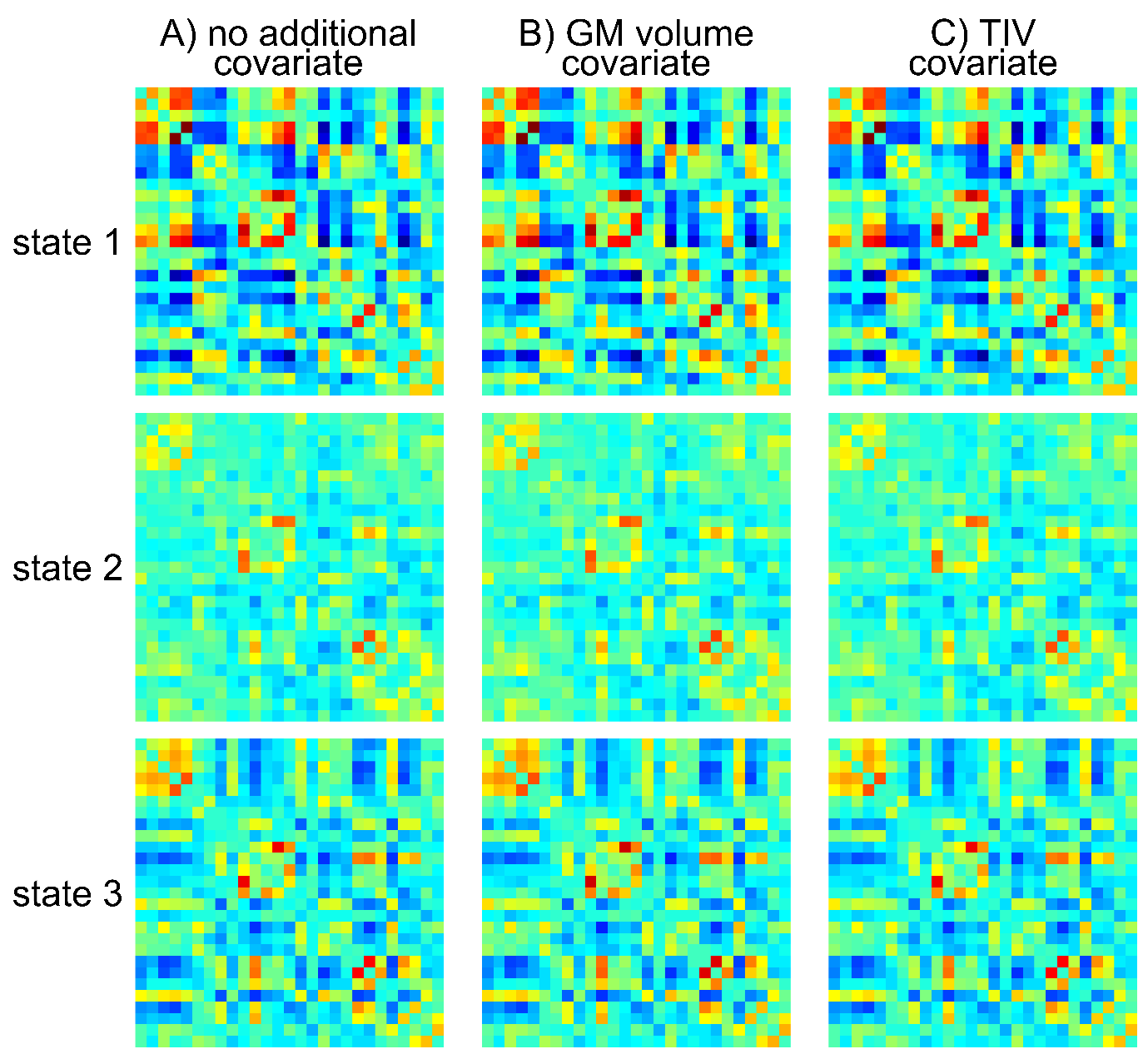


Figure S10: Effect of adding different covariates when residualizing the windowed correlation matrices (see Section 2.5 of the main text). A) K-means cluster centroids using only the covariates that were used in the main text (age, gender, study) for comparison (see Figure 3 of the main text). B) K-means cluster centroids after adding a covariate for total grey matter (GM) volume estimated by SPM. C) K-means cluster centroids after adding a covariate for total intracranial volume (TIV=GM volume+ white matter volume + CSF) estimated by SPM.
